# Reactions of Ions in Water Involving Halogen-Bonded Complexes

**DOI:** 10.1021/acscentsci.6c00682

**Published:** 2026-07-10

**Authors:** Imon Mandal, Mark A. Johnson, R. Benny Gerber

**Affiliations:** † The Fritz Haber Research Center for Molecular Dynamics, Institute of Chemistry, The Hebrew University of Jerusalem, Jerusalem 91904, Israel; ‡ Sterling Chemistry Laboratory, Department of Chemistry, 5755 Yale University, New Haven, Connecticut 06520, United States; § Department of Chemistry, University of California, Irvine, California 92697, United States

## Abstract

Reactions of ions in water are important in many areas of chemistry and biology, including the chemistry of seawater, atmospheric chemistry, and processes in proteins. Despite their importance, the microscopic mechanisms of these inherently complex and multistep reactions are mostly unknown so far. In this Outlook, we outline recent progress on atmospheric reactions of halogenated species with anions in water. These are found to proceed via halogen-bonded complexes, which are stabilized by the water environment. Such complexes were recently characterized experimentally in the gas phase with structures similar to those computed in water. We highlight two such reactions: one that leads to the formation of a dihalogen and is enhanced with acidity. The other reaction is of halogen exchange, which involves participation of water molecules that greatly catalyze the process. These findings suggest searches for many other reactions that may involve halogen-bonded complexes as precursors. On the basis of the present studies, searches for such reaction mechanisms can be effectively pursued by ab initio molecular dynamics (AIMD) simulations. The halogen-bonded complexes can be complementarily characterized by vibrational spectroscopy in mass spectrometry experiments.

## Graphical abstract

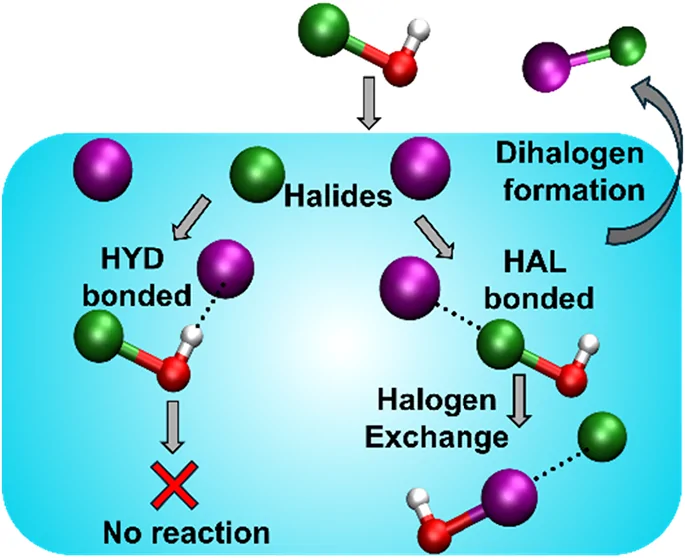

## 1 Introduction

Reactions of ions govern processes ranging from acid–base chemistry, redox reactions, catalysis, and energy conversion to biological ion transport and atmospheric reactions.^1–12^ Unlike neutral reactions, ion-driven processes due to the presence of charges, are controlled not only by local interactions but also by long-range electrostatics, solvent reorganization, and collective environmental effects. As a result, most aqueous ion reactions cannot be understood as simple bimolecular events, but instead emerge from cooperative, multistep processes involving ions, solvent molecules, and transient complexes. These effects become more pronounced at interfaces, where ions experience asymmetric solvation and altered electrostatic environments.^13–16^ Notably, ion-driven processes at the air-seawater interface have major impacts on the “chemistry of seawater”. An important class of such reactions is that involving interactions between halide ions present at the surface of water and incoming molecules from the gas phase. These reactions drive halogen activation and various cycles that process volatile organic and inorganic compounds and thereby influence tropospheric as well stratospheric oxidant budgets.^17–21^ While field observations, laboratory experiments, and atmospheric models have established the large-scale importance of these processes, their microscopic reaction pathways (particularly the details of the initial ion–molecule encounter and further processes) have remained largely unresolved until recently.

Here, AIMD^1–3,11,22,23^ simulations come to the fore, providing atomic detailing along with electronic evolution revealing that these reactions are governed by structured precursor complexes which organize incoming gas molecules with the ions at seawater. For halogenated gas-phase molecules reacting with halide ions in water, these precursors form a halogen-bonded complex between the ion and molecule. Such complexes are characterized in the gas phase experimentally by vibrational spectroscopy combined with mass spectrometry and other experimental techniques.^24,25^ AIMD simulations show that gas phase structures of these halogen-bonded precursor complexes are similar to the precursor structure in the presence of water, and these complexes stabilize reactive configurations, control the access to reaction pathways, and enable water-mediated catalytic mechanisms that differ fundamentally from gas-phase chemistry.^1–3,11^ In aqueous media, the cooperative role of water in mediating charge transport and catalyzing reactions is highlighted along with the influence of pH of the medium and presence of organic molecules on two such multistep atmospheric reactions: halogen exchange^2^ and dihalogen formation.^1,3^

This emerging view has profound implications for how ion-mediated reactions containing halogens proceed in water. This Outlook focuses on recent insights from AIMD simulations, vibrational spectroscopy in mass spectrometry experiments, and atmospheric chemistry at air–water interfaces as well as in the bulk to establish halogen bonding as a unifying mechanistic principle guiding reactions of ions in water. It will comprise the following sections:

The Outlook focuses on recent insights from AIMD simulations, vibrational spectroscopy in mass spectrometry experiments, and atmospheric chemistry at air–water interfaces as well as in the bulk to establish halogen bonding as a unifying mechanistic principle guiding reactions of ions in water.

## 2 Central Concept: Halogen-Bonded Precursors

A halogen bond (XB), as defined by International Union of Pure and Applied Chemistry, is a net attractive interaction between an electrophilic region on a halogen atom and a nucleophilic site in the same or another molecular entity. It typically forms when a halogen atom in dihalogens, interhalogens, or halogenated molecules interacts with a lone-pair-bearing nucleophile^26–29^ (Figure 1A). Due to their relatively high electronegativity, halogen atoms mostly behave as electron-rich (nucleophilic) sites and form net attractive interactions with electron-poor (electrophilic) partners. However, halogen atoms can also function as electron-poor (electrophilic) sites and attractively interact with electron-rich (nucleophilic) sites. The interaction arises from the anisotropic electron density around the halogen, which creates a positive electrostatic region known as a σ-hole which allows a nucleophile to approach and donate electron density into that region, stabilizing the complex. Figure 1B shows the electron density surrounding the I (iodine) which is covalently bound to an electronegative OH group is anisotropically distributed. Electron density is enhanced in the covalent bonding region between I and OH nuclei (i.e., over the cylindrical surface surrounding the bond), which results in negative electrostatic potential. In contrast, outside the I atom (around bond-end, blue and green region) electron density decreases resulting in positive electrostatic potential. This electropositive region commonly known as σ-hole participates in an attractive interaction with a nucleophile (here the halides). Same mechanism is valid for other HAL­[(HOX)···(Y^–^)]_aq_ (X,Y = halogens) complex formation. Upon further solvation by water molecules, the structured first solvation shell in Figure 1D is seen near the H of HOX. This is consistent with the electrostatic potential map indicating a positive electrostatic potential region near the H of the OH atom of the HOX. Halogen bonding can occur in gas, solution, and solid phases and is increasingly exploited in medicinal, supramolecular, and materials chemistry, as well as in catalysis and synthesis. Although the role of halogen bonding in chemistry is much less explored than hydrogen bonding, these bonds can play a major role in reactions.^1^ It was only in the past decade that the physics that underlies halogen bonding was clarified through computer simulations.^28^ These simulations predicted that an electron-withdrawing group attached to a halogen enhances σ-hole formation, and more polarizable halogens tend to form stronger halogen bonds.

**Figure 1:**
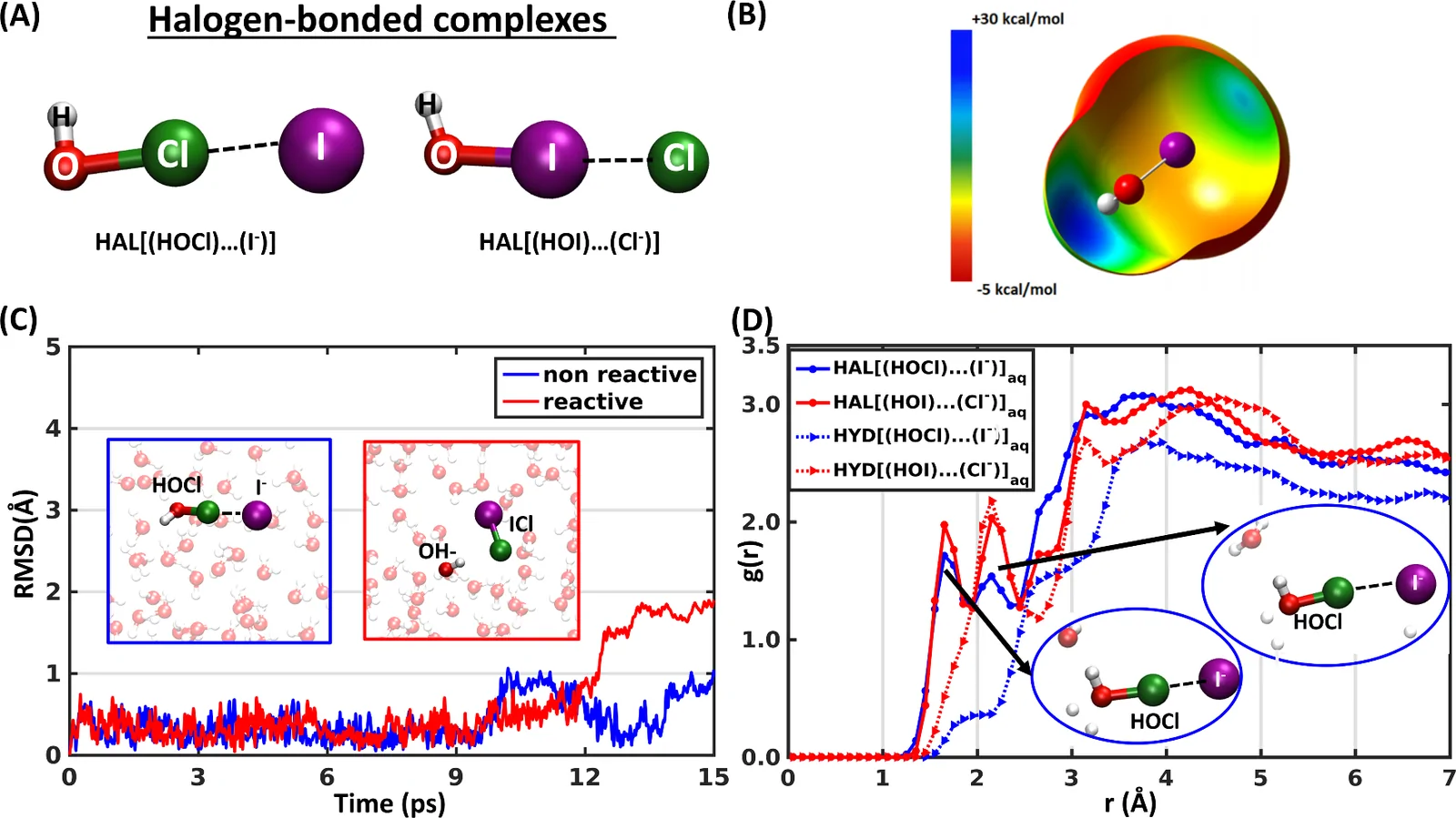
Stability of prereactive halogen-bonded complexes. (A) Gas phase optimized structure (MP2/aug-cc-pVTZ) of halogen-bonded prereactive complexes of [(HOX)···(Y^–^)] (X, Y = Cl, I or I, Cl). (B) Electrostatic potential map of the HOI molecule calculated at the MP2/aug-cc-pVTZ level on a 0.001 e/bohr^3^ surface. (C) RMSD of the HAL­[(HOCl)···(I^–^)]_aq_ complexes (four atoms H, O, Cl, and I) along the time trajectory shows the complexes are stable at least 15 ps except for the reactive ones. The nonreactive legend indicates one representative trajectory which remains unreactive during simulations. The reactive trajectory represents one of those that proceeds the halogen exchange reaction. Snapshots of the fate of the HAL­[(HOCl)···(I^–^)]_aq_ complexes at time 15 ps for the representative unreactive and reactive simulations are shown in the inset with colored boundaries. (D) Radial distribution plot of [(HOX)···(Y^–^)]_aq_ complexes with all other atoms present in the slab. Representative snapshots of 1st and 2nd solvation shell with water hydrogens and 1 oxygen of HAL­[(HOCl)···(I^–^)]_aq_ are provided in the inset. Reproduced from refs 2 and 24 . Copyright 2025, 2022 American Chemical Society.

This Outlook will advance the central concept that reactions of ions in water may proceed through halogen-bonded complexes which function as prereactive intermediates rather than through the more common hydrogen-bonded complexes.^1,2^ These halogen-bonded complexes exhibit directional charge transfer leading to reactions such as dihalogen formation from hypohalous acid (HOX, X = Cl, Br, I), nitryl halide (XNO_2_), or halogen nitrite (XONO) with halide ions (Y^–^, Y = Cl, Br, I) (eq 1–2).^1,3^ The halogen and hydrogen-bonded complexes are noted as HAL and HYD respectively, and the subscript aq is used to refer to the complexes in the presence of water.

$$HOX+Y^{-}\to \begin{array}{c} HAL{\left[\left(HOX\right)...\left(Y^{-}\right)\right]}_{aq} \end{array}\to XY+OH^{-}\;,,,,,,,,↓H^{+}\;,,,,,,,\begin{array}{c} XY+H_{2}O \end{array}$$ 1

2 $$XNO_{2}\;or\;XONO+2X^{‐}\to HAL[\left(XNO_{2}\right)...\left(X^{‐}\right){]}_{aq}\;or\;HAL[\left(XONO\right)...\left(X^{‐}\right){]}_{aq}+X^{-}\to X_{3}^{-}+NO_{2}^{-}\to X_{2}+X^{-}+NO_{2}^{-}$$

Reactions of ions in water may proceed through halogen-bonded complexes which function as prereactive intermediates rather than through the more common hydrogen-bonded complexes.

Several other atmospheric reactions such as the generation of Cl_2_O from hypochlorous acids,^30,31^ iodine halogen (I_2_, IBr, and ICl) formation from I_2_O_3_,^11^ and growth of new particles involving acid–base and hydration reactions in iodine oxoacid and oxide nucleation^22,23,32,33^ on aerosols, air–water interfaces, and bulk seawater are shown to process through a combination of halogen- and hydrogen-bonded complex precursors. However, in aqueous environments, collective solvent dynamics transform these microscopic motifs into catalytic reaction pathways, enabling efficient ion-driven chemistry, such as halogen exchange involving Grotthuss-like OH^–^ transport^2^ (*vide infra*).

Geometrical criteria are used to determine the formation and breaking of halogen bonds,^1,3,26^ e.g., for HAL­[(HOX)···(Y^–^)]_aq_ complexes (Figure 1A) a halogen bond is formed when the X–Y^–^ distance is smaller than 3.5 Å (1 Å = 1 × 10^–10^ m) and the ∠Y^–^···X–O angle is larger than 130°. The X–Y^–^/X^–^ distance distributions exhibit maxima below 3.0 Å, and ∠Y^–^/X^–^···X–O/N distributions peak ∼ 170° for halogen bonded complexes (e.g., HAL­[(XNO_2_)···(X^–^)]_aq,_ HAL­[(XONO)···(X^–^)]_aq_ and HAL­[(HOX)···(Y^–^)]_aq_). The root-mean-square deviation (RMSD) of the complexes depicts the variation of the behavior of the complexes in the presence of water from the reference initial structure (corresponding gas-phase species in Figure 1C). Stabilization in the RMSD of the complexes along trajectories signifies a stabilized complex structure. Here, Figure 1C indicates that HAL­[(HOCl)···(I^–^)]_aq_ complexes do not dissociate in the presence of a water slab for at least 15 ps (mentioned simulation time). This stability does not pertain to the trajectories where the reaction occurs, where after ∼4 ps the complexes can start reacting. The neighboring water molecules provide structural stabilization of the halogen bonded complexes. The radial distribution function (RDF) plots, denoted as *g*(*r*) in Figure 1D, of different [(HOX)···(Y^–^)]_aq_ complexes, which are proportional to the number of molecules within a spherical shell of radius r centered around the complex and averaged over all timeframes, reinforce this stabilization. Halogen-bonded complexes have two structured solvation layers below 3 Å indicated by two peaks, whereas the corresponding hydrogen-bonded complexes lack any structured solvation shell. These characterizations provide compelling evidence that halogen-bonded complexes persist for a sufficient duration in the presence of water, serving as crucial precursors to the atmospheric air–water interface or bulk reactions.

## 3 Water Can Act as a Catalyst in Reactions Involving Halogen-Bonded Complexes

AIMD simulations show that water does not merely screen electrostatic interactions but chemically participates in the reaction. Detailed atomistic mechanism of the halogen exchange reaction, HOCl + I^–^ → HOI + Cl^–^, by our group unraveled that this reaction proceeds via a Grotthuss-like hydroxide transport involving multiple water molecules catalytically at neutral pH—a stark contrast to the gas-phase mechanism.^2^ The qualitative mechanism involves three sequential steps: (1) ICl formation: HAL­[(HOCl)···(I^–^)]_aq_ → ICl + OH^–^, (2) migration of OH^–^ through a proton relay in water molecules by a Grotthuss-like mechanism: OH^–^ + H_2_O → H_2_O + OH^–^, (3) reaction of ICl and OH^–^ (attacking from the I side) to form the HAL­[(HOI)···(Cl^–^)]_aq_ complex (Figure 2). In some trajectories, the reaction stops with the formation of ICl, and OH^–^ migrating in the water slab but never reaches the I side of ICl during our simulation time scale (50 ps). ICl formation, the first step of the halogen exchange reaction, belongs to the highly important family of reactions in which halogens are formed from HOX and Y^–^. These reactions are by themselves of major atmospheric significance that occur under acidic conditions^34^ (*vide infra*). The variation observed here is that ICl is formed at neutral pH.

**Figure 2:**
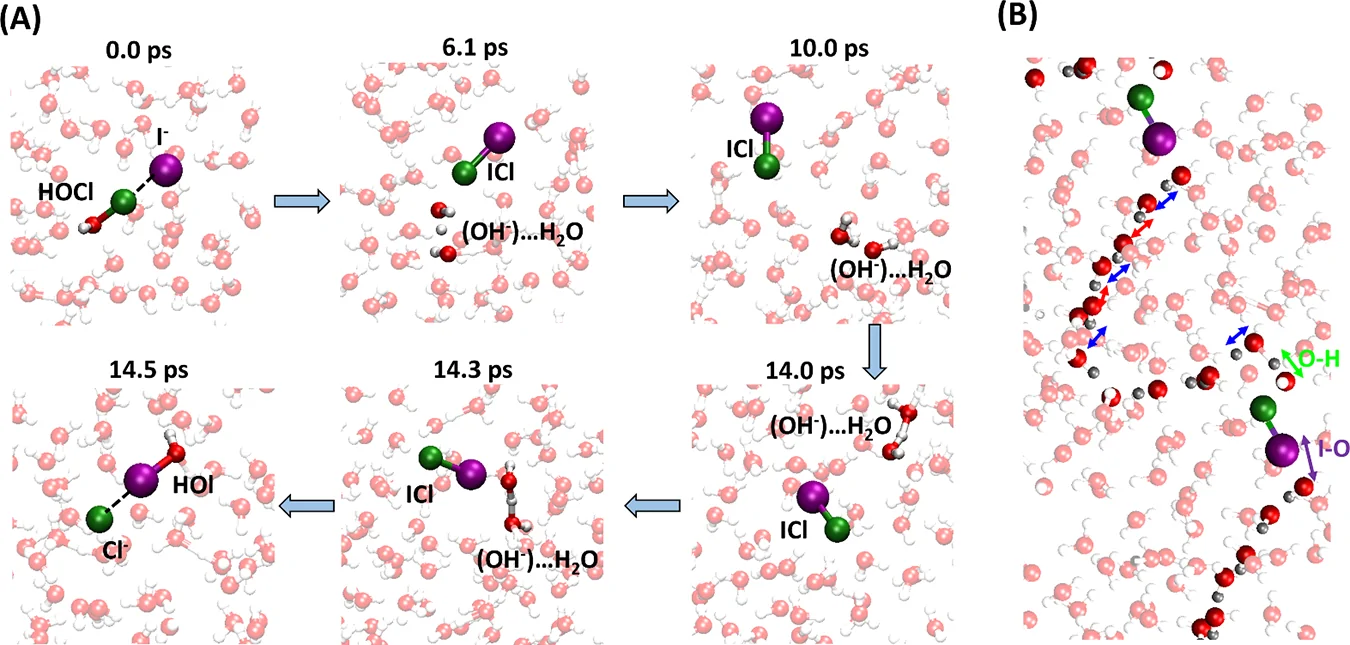
Halogen exchange reaction mechanism. (A) Snapshots for the multiple steps involved in the halogen exchange reaction HAL­[(HOCl)···(I^–^)]_aq_ to HAL­[(HOI)···(Cl^–^)]_aq_. (B) Snapshot of a representative trajectory of complete halogen exchange from HAL­[(HOCl)···(I^–^)]_aq_ to HAL­[(HOI)···(Cl^–^)]_aq_ showing all of the 12 water molecules involved in the Grotthuss-like OH^–^ transport. Reproduced from ref 2. Copyright 2025 American Chemical Society.

The whole process, from HAL­[(HOCl)···(I^–^)]_aq_ to HAL­[(HOI)···(Cl^–^)]_aq_, takes <50 ps (ranging from 1 to 12 ps, with an average of 4.3 ps, except 1 trajectory which takes 35 ps) and involves many water molecules ranging from 5 to 12 for the OH^–^ transfer. The chemical participation of water molecules leads to a much faster (by order of magnitude) halogen exchange than calculated to occur in the gas phase. The gas phase halogen exchange mechanism, HAL­[(HOCl)···(I^–^)] → HAL­[(HOI)···(Cl^–^)], involves three consecutive steps with a unimolecular rearrangement with half lifetimes ranging from microseconds to seconds at 300 K. Thus, water acts as a catalyst accelerating the reaction time from ms to ps for completion. Interestingly, the stable hydrogen-bonded complexes remain unreactive during simulations.

Cl_2_O generation from hypochlorous acid (HOCl) is catalyzed by one water molecule on aqueous/frozen interfaces and involves two elementary steps:^30^ (1) First HOCl molecule is deprotonated by water producing ClO^–^. (2) A second HOCl molecule participates in Cl^–^ abstraction by ClO^–^ leading to Cl_2_O formation. In the presence of water vapor in monomer, dimer, or trimer cluster forms, prereactive complexes are formed with a halogen bond between two HOCl molecules and one or more hydrogen bonds between HOCl and water or between water molecules. Cl_2_O is then subsequently generated within one step resulting from hydrogen atom transfer through the hydrogen bonding networks generated by water molecules. Cl_2_O generation from hypochlorous acid (HOCl) reacting with ClONO_2_ occurs with a lower energy barrier (50 times) in the presence of water clusters, air–water interface, and bulk liquid water through the formation of halogen-bonded HAL­[(ClONO_2_)···(HOCl)]_aq_ complexes^31^ than in the gas phase. Aerosol growth and interfacial hydration reactions of I_2_O_4_ in the presence of acid or base also becomes more favorable than the gas-phase reaction under ambient conditions due to the proton transfer along the “water bridge”.^22^

This solvent-driven cooperative catalytic effect of water mentioned above for different reactions becomes especially pronounced with with high water content characteristic of seawater and atmospheric aerosols rather than water vapor. These reactions are sometimes also catalyzed by acid (e.g., nitric acid, sulfuric acid, etc.) and base (e.g., ammonia, amine, etc.).^22,30^

## 4 Role of pH in Reactions

The pH of the environment/medium has a significant effect on the fate of the chemical reactions and affects the tropospheric multiphase oxidant budget enormously.^1,35,36^ Atmospheric Cl_2_ formation proceeds through halogen-bonded complexes of HAL­[(HOCl)···(Cl^–^)]_aq_ at the air–water interface and undergoes charge transfer from Cl^–^ to OH^–^ to form transient Cl_2_ at neutral pH. AIMD simulations with addition of a proton to one proximal water forming hydronium ion (H_3_O^+^) greatly facilitates the Cl_2_ formation.^1^ Indeed, this proton addition mimicking the acidity of the water medium or the low pH experimental condition stabilizes the charge on OH^–^ forming neutral H_2_O (water). Here, Cl_2_ formation is nonreversible (e.g., does not revert to the HAL­[(HOCl)···(Cl^–^)]_aq_ complex) and escape from the water slab (Figure 3). This enhancement of the Cl_2_ formation reaction in acidic environments is supported by laboratory experiments^37,38^ and field observations.^36,39^ Furthermore, the specific orientation of the nearest proton of the hydronium ion to the OH group of HOCl is essential for the reaction to proceed. The optimal angle to be formed between the OH and the excess proton during the process is found to be in the range ∼80–120°. Similarly, HOI reacts with the interfacial I^–^ ions through the halogen-bonded reaction complex HAL­[(HOI)···(I^–^)]_aq_ formed by the attraction between the electron-rich I^–^ ion and the electron-deficient σ-hole of HOI.^11^ This interfacial reaction is also affected by a proximal H_3_O^+^ ion, showing that proton from H_3_O^+^ rapidly transfers along the water bridge, finally yielding 2H_2_O + I_2_. We also observed similar Br_2_ formation when HAL­[(HOBr)···(Br^–^)]_aq_ complexes are simulated with addition of an excess proton as in ref 1. Not only dihalogens, but interhalogens like ICl, are also formed under such acidic conditions.^2,34^ In these cases, no further reaction occurs after HAL­[(HOCl)···(I^–^)]_aq_ + H^+^ → ICl + H_2_O. These results indicate that the all dihalogen (X_2_) and interhalogen (XY) formation proceeds rapidly on acidified ocean or aerosol surfaces through halogen bonded [(HOX)···(X/Y)^−^]_aq_ complexes.

**Figure 3:**
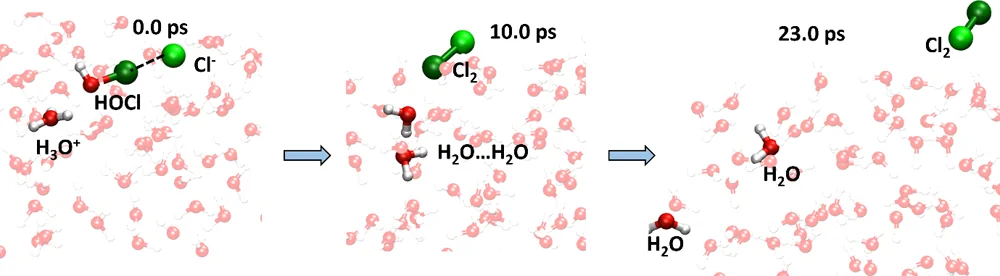
Snapshots of the HAL­[(HOCl)···(Cl^–^)]_aq_ complexes with hydronium ion (H_3_O^+^) at different timesteps. Reproduced from ref 1. Copyright 2024 American Chemical Society.

In another pathway, I_2_ formation from I_2_O_3_ reacting with the amines is also affected by environmental pH.^11^ Amines are likely to be protonated in acidic medium (pH < 7) and become unable to react with I_2_O_3_. At seawater surface (pH = ∼8) amine-mediated reaction takes place through a prereactive complex with halogen and hydrogen bonds forming HOI and IO_3_^–^ and subsequently I_2_.

## 5 Surface versus Bulk Reactivity and the Effect of Organics

At air–water interfaces, product release becomes more facile than the bulk water. This provides the expectation for efficient dihalogen production from reactions of hypohalous acids with halide ions at the seawater interface (Figure 3) and sea-salt aerosols—key steps in atmospheric halogen activation. Our new results with the gas phase optimized [(HOX)···(Y^–^)] species in a cubic water box (12.42 Å length) of 64 water molecules show that halogen exchange and dihalogen formation mechanisms remain the same in the bulk as at the interface (Figures 2 and 3). We also observe that addition of organic components (e.g., alcohol molecules) does not alter these mechanisms. A water slab model with unit cell made of 70 water molecules, 1 methanol, and 1 gas phase optimized [(HOCl)···(I^–^)] complex in a 13.47 × 15.56 × 40 Å^3^ rectangular box was used in these AIMD simulations. The concentration of methanol in the box is ∼0.8 M, higher than the seawater concentration of ∼100 nM. Thus, 1 methanol should be enough for this slab to observe if alteration occurs in the dihalogen formation or halogen exchange mechanism from the reactions of hypohalous acids with halide ions. All other details of the simulations are same as mentioned in refs 1 and 2. Even in the presence of methanol, both ICl formation and halogen exchange proceed through halogen-bonded HAL­[(HOCl)···(I^–^)]_aq_ complexes. ICl formation is facilitated by the presence of an excess proton, and formation of HAL­[(HOCl)···(I^–^)]_aq_ → HAL­[(HOI)···(Cl^–^)]_aq_ occurs via chemical participation of water through Grotthuss-like OH^–^ transport (Figure 4). However, in some trajectories, methanol also participates in the proton relay mechanism of the OH^–^ transport (Figure 4A, 24.3 ps snapshot).

**Figure 4:**
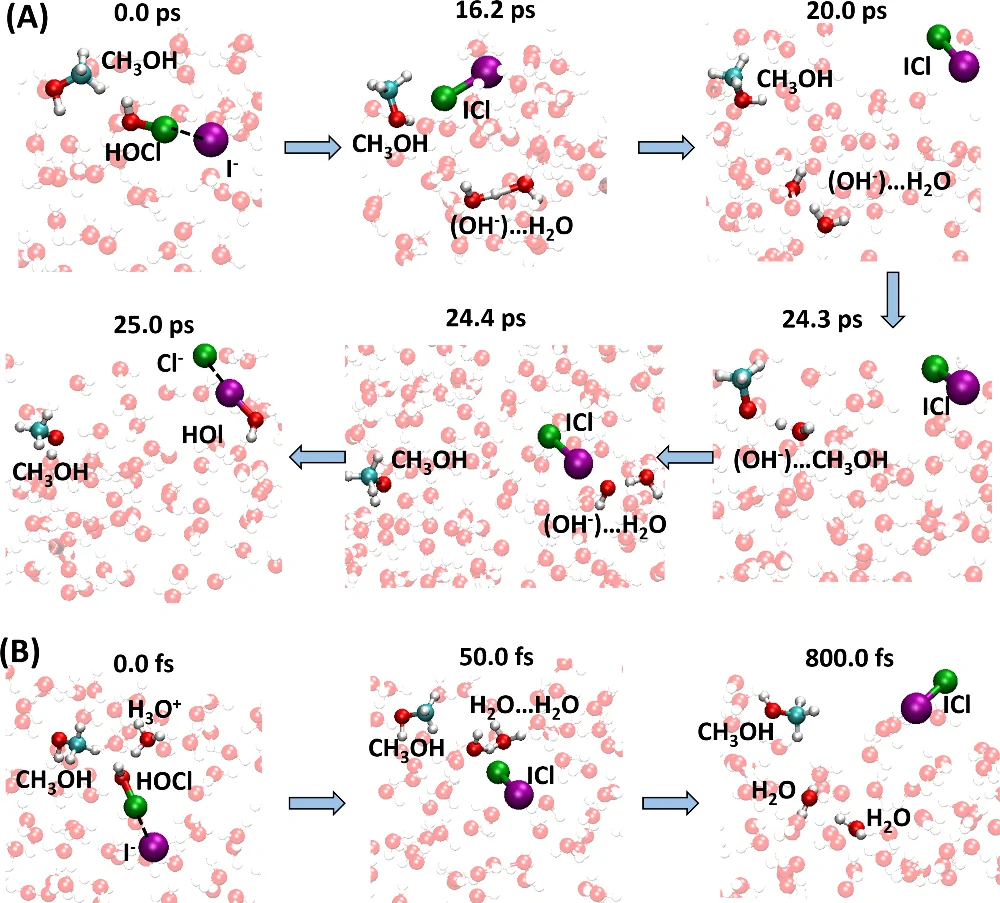
Halogen exchange and ICl formation mechanism in the presence of methanol. (A) Snapshots for the multiple steps involved in the halogen exchange reaction HAL­[(HOCl)···(I^–^)]_aq_ to HAL­[(HOI)···(Cl^–^)]_aq_ in the presence of one methanol. (B) Snapshot of a representative trajectory of ICl formation with hydronium ion (H_3_O^+^) at different timesteps.

## 6 Methodological Perspective: Theory

Another theme of this Outlook is the necessity of explicit-solvent, dynamics-based approaches. Static electronic-structure calculations and continuum solvation models are fundamentally unable to capture the formation, persistence, and reactivity of halogen-bonded precursor complexes. AIMD offers a powerful and general strategy for identifying charge transfer reaction gateways in aqueous ion chemistry.^1–3,11,22,23^ For example, atomistic details of reactions like dihalogen/interhalogen formation from hypohalous acid with halide ions (which proceeds through halogen -bonded complexes not hydrogen-bonded ones) were investigated using DFT-based AIMD simulations.^1,2,11^ However, gas phase static calculations show HAL­[(HOX)···(Y^–^)] is less stable than HYD­[(HOX)···(Y^–^)] (except [(HOI)···(Cl^–^)]). In the presence of a water slab, the HAL­[(HOX)···(Y^–^)]_aq_ complexes are stabilized by the water envelope (Figure 1D) and enable the reaction. The molecular mechanism of the halogen exchange reaction, HOCl + I^–^ → HOI + Cl^–^, can also be unraveled using DFT-based AIMD. The reaction proceeds via a Grotthuss-like hydroxide transport involving catalysis by multiple water molecules, an insight that cannot be obtained from static calculations.^2^ Multistep dihalogen formation from nitryl halide (XNO_2_) or halogen nitrite (XONO) with halide ions,^3^ atmospheric iodine halogens (I_2_, IBr and ICl) formation from I_2_O_3_,^11^ and growth of new particles of iodine oxoacids and iodine oxides involving acid–base and hydration on aerosol, water droplets or air–water interfaces^22,23,32,33^ were also investigated using AIMD simulations. AIMD simulations not only provide evolution of atomic nuclei in time but also electronic properties like partial charges of atoms, orbital localization, and delocalization along the simulation trajectory, thus providing detailed insights and interpretations on the processes delocalized over various molecular entities.^40,41^

AIMD simulations not only provide evolution of atomic nuclei in time but also electronic properties like partial charges of atoms, orbital localization, and delocalization along the simulation trajectory, thus providing detailed insights and interpretations on the delocalized processes.

## 7 Methodological Perspective: Experiment

Many of the experiments that are central to the Outlook were carried out in bulk water, or at the air-water interface. Particularly important microscopic insights were obtained in the study of gas-phase clusters relevant as models for the bulk systems.^24,25^ Insights into the halogen exchange reaction HOCl + I^–^ → HOI + Cl^–^ were provided by exploring the structure and stability of dry gas-phase complexes of [(HOCl)···(I^–^)]. The HAL­[(HOI)···(Cl^–^)] complex was characterized, which formed upon uptake of HOCl vapor by I^–^·(H_2_O)_*n*_ clusters in a radiofrequency ion trap and characterized by analysis of the vibrational band pattern.^24^ However, the role of water molecules was left unexplored, leaving open questions such as whether it was chemically active in this process or simply served as a third body to stabilize the collision complex. Confirmation of chemical participation of water was obtained from AIMD simulations.^2^ Another example of a halogen -bonded complex of halide ions is the HAL­[(Br^–^)···(BrCCl_3_)] complex, which was observed and characterized using photoelectron spectroscopy.^42^ Other experimental techniques which have provided useful information about the halogen bonding include molecular beam scattering,^43^ ion-mobility measurements,^44^ blackbody infrared radiative dissociation,^45^ and rotational spectroscopy.^46^

## 8 Outlook

This Outlook establishes halogen-bonded complexes as central reaction precursors governing ion reactivity with incoming gas molecules at seawater, bridging insights from theoretical simulations and spectroscopy to seawater chemistry, atmospheric chemistry, and beyond. By providing a unified mechanistic framework that links microscopic electronic structure calculations to macroscopic chemical outcomes, we aim to offer a perspective that will be broadly useful to researchers across physical chemistry, atmospheric chemistry, and aqueous electrolyte chemistry. Our findings suggest new pathways may be in play for many other reactions in water (especially involving halide ions) which involve halogen-bonded complexes as precursors. Recent reports show that halogen-bonded complexes are used as a reactive precursor motif in organocatalysis,^4,5^ photochemical activation,^6,47^ biological systems such as protein–ligand interaction, drug design, enzymatic catalysis,^48^ and medicinal chemistry.^49^ We suggest further studies of the possible role of halogen bonding in reactions of atmospheric chemistry, biological chemistry, and aqueous electrolysis. In fact, the role of the halogen-bonded precursors remains largely unexplored in energy-related processes (e.g., battery designing, electrolyte chemistry) and represents a promising avenue for future research. For example, halogen-bonded complexes may be involved in the electrocatalytic chlorine evolution reactions (CER) on Pt-based catalysts showing remarkable selectivity over the competitive oxygen evolution reactions (OER).^50^ The studies discussed here suggest that AIMD simulations are effective computational methods to explore new reaction mechanisms. Experimentally, vibrational spectroscopy in mass spectrometry experiments could be used for better understanding of the halogen-bonded complexes in water as those are the same as gas phase structures.

We suggest further studies of the possible role of halogen bonding in reactions of atmospheric chemistry, biological chemistry, and aqueous electrolysis.

## Supplementary Material



## Notes

Transparent Peer Review report available (PDF)
