# Supplementary material for: Reactions of Ions in Water Involving Halogen-Bonded Complexes
Source: ACS Cent Sci. 2026 Jul 10;12(9):1287–94. doi: 10.1021/acscentsci.6c00682 (PMC13626023; doi:10.1021/acscentsci.6c00682)
Supplement: Supplementary file 1 [file oc6c00682_si_001.pdf]

Responses to the reviewers and revisions made

Dear Editor,

Thank you for conveying the comments of the reviewers to us. We thank all the reviewers for providing very encouraging comments and finding our central concept of halogen bonding complexes acting as reaction precursors very interesting. We thank reviewer 1 and reviewer 3 for offering new perspectives on the potential applications of our halogen bonded reaction precursors in chemical biology and material science fields.

We have revised the paper in response to the comments of the reviewers and have attempted to address their concerns. We believe this has indeed improved the quality of the paper. Below, we give our detailed responses to all comments of the reviewers, and outline the revisions made for each point. We have also provided proper copyright permissions for Figure 1, Figure 2 and Figure 3 (highlighted in yellow).

Reviewer: 1

Reviewer's Summary:
I am reviewing this manuscript as an outsider to its direct technical aspects; rather, this review provides a perspective on whether this Outlook will have "broad interest to other researchers". My feeling is that organic, supramolecular, and physical organic chemists will find this interesting even if they might not dive into the paper deeply. Given the wide use of halogen bonding in anion recognition, and emerging uses in chemical biology and catalysis, the finding that halogen bonding complexes are central reaction precursors for a variety of reactions in or on water is quite interesting. I would not say that finding is surprising to me; however, providing strong computational and experimental evidence for a few key reactions is needed to raise the importance of these types of interactions in mechanistic chemistry. Therefore, I think this paper could rise to the level of broad interest required in ACS Central Science, and the authors provide important guidelines for others working in this area to make sure they use the right computational and spectroscopic approaches. It is worth noting that I don't believe the authors wrote this Outlook looking to appeal to researchers in my field. They write that "we aim to offer a perspective that will be broadly useful to researchers across physical chemistry, atmospheric chemistry, and aqueous electrolyte chemistry". So researchers in those fields should be consulted, but perhaps it is helpful to know that at least one researcher in my field enjoyed learning about this observation of halogen bonding in some aqueous ion reactions.

Authors Response: We thank the reviewer for this thoughtful and encouraging assessment of our work. We appreciate the recognition that the identification of halogen-bonding complexes as key reaction precursors in aqueous environments may have broad implications in organic, supramolecular, and physical organic chemistry. We highly value the reviewer's perspective on the emerging implications of halogen

bonding in areas such as chemical biology and catalysis, as well as the potential of our study to provide useful methodological guidelines for future investigation combining robust computational and experimental spectroscopic approaches.

Reviewer's comment 1: At the start of section 2, the authors summarize the IUPAC definition of a halogen bond and say it "is a non-covalent attractive interaction...". I would replace non-covalent with "net" to be consistent with the definition (which does not restrict the attraction to being only non-covalent).

Authors reply: We agree with the referee's viewpoint. Halogen bond is a combination of non-covalent electrostatic, exchange repulsion, inductive, dispersion and covalent interactions and the strength of the bond is determined by the interplay of these interactions. In our systems of $[(HOX)\ldots(Y^{-})]_{aq}$ (X,Y=halogens) we believe the halogen complexes are mostly interacting by non-covalent electrostatic interactions, however further detailed energy decomposition calculations are required to obtain the decomposition of the various interactions which is outside the scope of this Outlook article.

Revisions made: In the revised version of the manuscript, we modified the IUPAC definition: "A halogen bond (XB), as defined by International Union of Pure and Applied Chemistry, is a net attractive interaction between an electrophilic region on a halogen atom and a nucleophilic site in the same or another molecular entity." (highlighted in yellow).

Reviewer's Comment 2: At the tope of page 7, there are distance and angle cutoffs listed for halogen bonds to occur (X-Y- distance < 3.5A, for instance). Where do these numbers come from? They are not in the IUPAC definition. Furthermore, the community is moving away from distance and angle cutoffs in defining these types of attractive interactions. Perhaps providing average distances and angles for bona fide examples of these interactions is more suitable here.

Authors reply: According to IUPAC recommendations (IUPAC Recommendations 2013) "the interatomic distance between X and the appropriate nucleophilic atom of Y tends to be less than the sum of the van der Waals radii, X is any halogen atom with an electrophilic region and Y is the halogen bond acceptor and is typically a molecular entity possessing at least one nucleophilic region". Thus, we choose 3.5 Å which is the summation of two chlorine atoms van der Waals radii (smallest halogen) as our limiting halogen bond formation criteria for all $[(HOX)\ldots(Y^{-})]_{aq}$ (X,Y=halogens). In the next sentence we provided the distance and angle distribution data with the average distances and angles as mentioned by the reviewer. We modified the sentence in the revised manuscript to clarify this. Our viewpoint is also consistent with the referee's note that "the community is moving away from distance and angle cutoffs in defining these types of attractive interactions". Thus, we reported root-mean-square deviations (RMSD) of the structures of the complexes as measure of stability along the simulation trajectory.

Revisions made: We modified the sentence at the bottom of page 7 in the revised version of the manuscript, to clarify our point of the average of distance and angle distributions: "The X−$Y^−$/$X^−$ distance distributions exhibit maxima below 3.0 Å and ∠$Y^−$/$X^−$···X−O/N distributions peak ~170° for halogen bonded complexes (e.g. HAL[(XNO_2)…(X^-)]$_{aq,}$ HAL[(XONO)…(X^-)]$_{aq}$ and HAL[(HOX)…($Y^−$)]$_{aq}$). (highlighted in yellow). We also highlighted in yellow the next section which describes root-mean-square deviation as measure of stability of the halogen bonded complexes.

Reviewer: 2

Reviewer's Summary:
This Outlook article explores the role of halogen-bonded (XB) complexes as pre-reactive intermediates in aqueous ion chemistry, specifically focusing on atmospheric reactions at the air-seawater interface. The authors utilize Ab-Initio Molecular Dynamics (AIMD) simulations to demonstrate how these complexes are stabilized by the water environment and how water acts as a chemical catalyst through Grotthuss-like mechanisms. The topic is of high current interest, bridging microscopic electronic structure with macroscopic atmospheric outcomes. The methodology is sound, and the results provide significant insights into halogen activation cycles.

Authors Response: We thank the reviewer for these valuable remarks and pointing out textual inconsistencies. We have addressed all these issues and highlighted the modifications in yellow.

Reviewer's Comment 1: In the description of Equation 1, the text mentions "hypohalous acid (HOX, Y=Cl, Br, I)"; it should be "HOX (X=Cl, Br, I)".

Authors reply and revisions made: We changed (HOX, Y=Cl, Br, I) to (HOX, X=Cl, Br, I) in the description of Equation 1.

Reviewer's Comment 2: Equation 2 is not balanced, please correct the stoichiometry

Authors reply and revisions made: We corrected the stoichiometry of Equation 2.

Reviewer's Comment 3: On page 7, the text states the complexes do not dissociate for "at least 15 ps". However, in the section regarding halogen exchange, the average time is cited as "4.3 ps" with a range up to "35 ps" or "50 ps". PLease check if the the "15 ps" stability claim in Section 2 should be modified.

Authors reply and revisions made: We apologize for the confusion. On page 8 in the revised manuscript "Here, in Figure 1C indicates that HAL[(HOCl)…($I^−$)]$_{aq}$ complexes do not dissociate in presence of water slab for at least 15 ps" this sentence indicates that halogen-bonded complexes are stabilized for at least 15ps. However, this stability only pertains to non-reactive trajectories. Reactive trajectories can start proceeding the reactions after 4ps. We added a line on page 8 of the revised manuscript to clarify this:

"This stability does not pertain to the trajectories where the reaction occurs, there after ~4ps the complexes can start reacting." (highlighted in yellow).

Reviewer's Comment 4: The subscript "aq" is used inconsistently. That is, it is used in come parts of the text but often omitted in some figure captions or other parts of the text for the same species.

Authors reply and revisions made: We corrected these inconsistencies. In the manuscript, subscript aq is used to refer to the complexes in presence of water and when subscript aq is not used, the notation refers to the gas-phase complexes. Thus, Fig 1A caption will remain as [(HOX)…(Y^-)] which depicts gas phase optimized structures. Fig 1D caption "distribution plot of [(HOX)…(Y^-)] complexes" has been modified to "Radial distribution plot of $[(HOX)\ldots(Y^-)]_{aq}$ complexes" in the revised manuscript (highlighted in yellow). On Page 14 of the revised manuscript, "However, gas phase static calculations show HAL$[(HOX)\ldots(Y^-)]_{aq}$ is less stable than HYD$[(HOX)\ldots(Y^-)]_{aq.}$ (except $[(HOI)\ldots(Cl^-)]_{aq}$)" is modified to "However, gas phase static calculations show HAL[(HOX)…(Y^-)] is less stable than HYD[(HOX)…(Y^-)]. (expect [(HOI)…(Cl^-)])." (highlighted in yellow).

Reviewer: 3

Reviewer's Summary:
The authors provide an insightful outlook on the role of halogen-bonded (HAL) complexes as critical pre-reactive intermediates in aqueous and atmospheric chemistry. Using AIMD simulations, the authors demonstrate that reactions between halogenated gas-phase molecules (e.g., HOX, XNO2) and halide ions at the air-water interface do not follow traditional hydrogen-bonding pathways. Instead, they proceed through structured HAL complexes characterized by electrophilic halogen-halide interactions. A particularly compelling finding is the catalytic role of water, which facilitates halogen exchange through a Grotthuss-like OH- transport mechanism involving multiple water molecules. The use of AIMD effectively captures the transient nature of HAL complexes and the specific participation of water molecules in the chemical transformation—details often lost in static or gas-phase models. Furthermore, the observation that water molecules directly catalyze halogen exchange at neutral pH provides a new perspective on how solvent environments actively participate in "solvent-mediated" chemistry. The authors successfully establish halogen bonding (XB) as a "unifying mechanistic principle" for ion-mediated reactions in water. The significance of this work is amplified when viewed through the lens of fundamental chemical physics and practical industrial applications. This manuscript presents a robust and theoretically sound argument for the importance of halogen bonding in aqueous ion reactions. It is recommended for publication following revisions that further integrate the physical-chemical origins of anisotropic interactions and their broader implications for halogen activation.

Authors Response: We thank the reviewer for these thoughtful remarks.

Reviewer's Comment 1: Physical Origin of Interactions
The manuscript's focus on "anisotropic electron density" is deeply consistent with established research regarding the \sigma-hole—a region of electron deficiency at the halogen bond-end that allows these atoms to interact with anions despite their overall electronegativity. As previously elucidated in the literature (e.g., Sci. Rep. 4, 5826), the anisotropic charge distribution and anisotropic van der Waals radii of group VII elements are the primary drivers for such noncovalent interactions. This fundamental framework provides the necessary physical justification for the stability of the aqueous HAL complexes (lasting ~15 ps) reported in this study. The authors should:

- Elaborate on this anisotropy or more explicitly link their observations of "structured solvation shells" to the anisotropic van der Waals radii of the halogen atoms.
- Discuss how the inversion of anisotropy (the potential difference between the bond-end and the cylindrical bond surface) stabilizes these complexes to strengthen the "Central Concept" section.
- Briefly discuss how these intermediates might behave in more confined or heterogeneous environments, such as near the surface of N-doped graphene or other catalytic frameworks where local electronic environments vary significantly.

Authors reply: We thank the reviewer for drawing attention to an additional perspective on the physical origin of halogen bonding and for providing the relevant reference. As noted by the referee very correctly that "the anisotropic charge distribution and anisotropic van der Waals radii" of the halogens are primary drivers of halogen bonding non-covalent interaction's part. In the present manuscript in Figure 1B, the halogen bonding between HOI with halides is characterized by using an electrostatic potential map of HOI. Electron density surrounding the I(iodine) which is covalently bound to an electronegative OH group is anisotropically distributed. The electron density enhances in the covalent bonding region between I and OH nuclei (i.e., over the cylindrical surface surrounding the bond), which results in negative electrostatic potential. In contrast, outside the I atom (around bond-end, blue and green region) electron density decreases resulting in positive electrostatic potential. This electropositive region commonly known as "σ-hole" participate in an attractive interaction with a nucleophile (here the halides). Same mechanism is valid for other HAL[(HOX)…(Y^-)]$_{aq}$ (X,Y=halogens) complex formation.
Regarding the anisotropic van der Waals' radii of halogen atoms, the electrostatic potential map suggests that radii of halogens (here iodine in Figure 1B) would be smaller along the bond axis than in the perpendicular direction. Thus, the halogen bond formation will happen at the bond end. Upon further solvation by water molecules, structured 1st solvation shell in Figure 1D is seen near the H of HOX. This is consistent with the electrostatic potential map indicating positive electrostatic potential region near H of the OH atom of the HOX. This solvation pattern facilitates both dihalogen

formation and halogen-exchange reactions. Overall, we emphasize mainly the anisotropic charge distribution to explain the halogen bonding as we believe the halogen complexes are predominately interacting by this non-covalent electrostatic interaction. We added few sentences in Section 2 of the revised manuscript to clarify our central concept. Moreover, the primary objective of our work is to identify and characterize the halogen-bonded complexes that act as precursors to subsequent chemical reactions; thus, we did not go further with the decomposition of various covalent and non-covalent energy components of halogen bonding.

From the overall Outlook, it is very clear that local environment will have huge effects on the reactivity of these halogen-bonded reaction precursors. For example, halogen exchange reaction $HOCl+I^- \rightarrow HOI+Cl^-$ at air-water interface region which proceeds through $HAL[(HOCl)\ldots(I^-)]_{aq}$ via Grotthuss-like hydroxide transport at neutral pH. This mechanism changes totally in presence of excess proton in water formatting ICl from the same $HAL[(HOCl)\ldots(I^-)]_{aq}$ precursors. However, these mechanisms do not change in the bulk of the seawater or presence of organics. Thus, we can predict that heterogeneous environments, such as near the surface of N-doped graphene or other catalytic frameworks where local electronic environments vary significantly, the stability of the precursors will be different depending on the position of attachment and reaction mechanism can also alter. However, without detailed AIMD simulations of a specific set of reactions, it is very challenging to comment on the exact behavior of these halogen bonded complexes. We explained the effect of local environments in *Section 4. Role of pH in reactions* and *5. Surface versus bulk reactivity and the effect of organics* for some reactions which proceeds through halogen-bonded complexes.

Revisions made: We added the reference in our revised manuscript as Ref 29 (highlighted in yellow). We added on Section 2, page 5 of the revised manuscript "Figure 1B shows the electron density surrounding the I(iodine) which is covalently bound to an electronegative OH group is anisotropically distributed. Electron density enhances in the covalent bonding region between I and OH nuclei (i.e., over the cylindrical surface surrounding the bond), which results in negative electrostatic potential. In contrast, outside the I atom (around bond-end, blue and green region) electron density decreases resulting in positive electrostatic potential. This electropositive region commonly known as "σ-hole" participate in an attractive interaction with a nucleophile (here the halides). Same mechanism is valid for other $HAL[(HOX)\ldots(Y^-)]_{aq}$ (X,Y=halogens) complex formation. Upon further solvation by water molecules, structured 1st solvation shell in Figure 1D is seen near the H of HOX. This is consistent with the electrostatic potential map indicating positive electrostatic potential region near H of the OH atom of the HOX." (highlighted in yellow) to strengthen our central concept.

Reviewer's Comment 2: Industrial and Environmental Relevance & Broadening the Outlook

The mechanistic insights into halogen activation and chlorine species (Cl2) carry

profound implications for the design of high-efficiency electrocatalysts. For instance, in the development of single-atom catalysts (SACs) for the chlorine evolution reaction (ClER), understanding these exchange mechanisms is vital for achieving the near 100% selectivity and extraordinary mass activity recently demonstrated in Pt-based -C2N2 systems (Small 19, 2207128). The authors could bridge the gap between fundamental atmospheric pathways and the optimization of industrial halogen production. A brief discussion on how these HAL precursors might influence the selectivity of industrial processes (specifically the competition between ClER vs. OER) would significantly enhance the manuscript's impact on the broader chemical and materials science communities.

Authors reply: We thank the reviewer for this insightful comment and for highlighting the broader industrial relevance of our work. We agree that bridging the gap between fundamental atmospheric pathways and industrial halogen production can significantly broaden the impact of our manuscript.
In this Outlook article, we mainly discussed atmospheric reactions of ions in water that proceed via halogen bonded complexes elucidating the formation, stability and reactivity under different environmental conditions. Nevertheless, the obtained mechanistic insights may also be relevant to electrocatalytic chlorine production, where the stabilization and transformation of chlorine-containing intermediates play a crucial role in determining catalytic activity and selectivity as noted by the reviewer in a reference (we are not able to find that reference, so added Ref 50 with similar chlorine evolution reaction mechanism, CER). In particular, the selective formation and activation of chlorine-containing species at anode interfaces are central to promoting the chlorine evolution reaction while suppressing the competing oxygen evolution reaction (OER). Our Outlook suggests that how halide ions interact with electrophilic halogen-containing species. In CER the reactions of Cl attached to the Pt-based catalyst can react through same mechanism to the chloride available more than to the oxygen favouring CER over OER. To acknowledge this broader perspective, we have added a sentence in *Section 8 Outlook* of the revised manuscript mentioning the potential relevance of our findings to chlorine evolution chemistry.

Revisions made: We added "For example, halogen-bonded complexes may be involved in the electrocatalytic chlorine evolution reactions (CER) on Pt-based catalyst showing remarkable selectivity over the competitive oxygen evolution reactions (OER)." in *Section 8 Outlook* of the revised manuscript (highlighted in yellow).

We hope that with the revisions we made, and in view of the very supportive comments of all reviewers, you will decide to accept the paper. We look forward to your decision.

Sincerely,

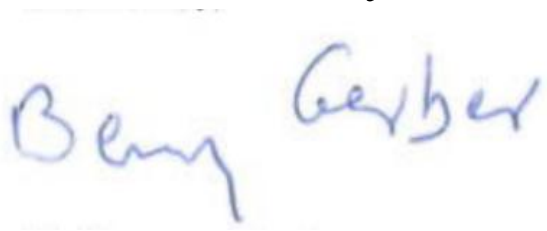

Robert Benny Gerber
On behalf of all co-authors
